# Diet and lifestyle impact the development and progression of Alzheimer’s dementia

**DOI:** 10.3389/fnut.2023.1213223

**Published:** 2023-06-29

**Authors:** Sarah Arora, Jose A. Santiago, Melissa Bernstein, Judith A. Potashkin

**Affiliations:** ^1^Center for Neurodegenerative Diseases and Therapeutics, Cellular and Molecular Pharmacology Discipline, The Chicago Medical School, Rosalind Franklin University of Medicine and Science, North Chicago, IL, United States; ^2^NeuroHub Analytics, LLC, Chicago, IL, United States; ^3^Department of Nutrition, College of Health Professions, The Chicago Medical School, Rosalind Franklin University of Medicine and Science, North Chicago, IL, United States

**Keywords:** Mediterranean diet, MIND diet, DASH diet, ketogenic, dementia, Alzheimer’s disease, type 2 diabetes, intermittent fasting

## Abstract

Dementia is a growing public health concern, with an estimated prevalence of 57 million adults worldwide. Alzheimer’s disease (AD) accounts for 60–80% of the cases. Clinical trials testing potential drugs and neuroprotective agents have proven futile, and currently approved drugs only provide symptomatic benefits. Emerging epidemiological and clinical studies suggest that lifestyle changes, including diet and physical activity, offer an alternative therapeutic route for slowing and preventing cognitive decline and dementia. Age is the single most common risk factor for dementia, and it is associated with slowing cellular bioenergetics and metabolic processes. Therefore, a nutrient-rich diet is critical for optimal brain health. Furthermore, type 2 diabetes (T2D) is a risk factor for AD, and diets that reduce the risk of T2D may confer neuroprotection. Foods predominant in Mediterranean, MIND, and DASH diets, including fruits, leafy green vegetables, fish, nuts, and olive oil, may prevent or slow cognitive decline. The mechanisms by which these nutrients promote brain health, however, are not yet completely understood. Other dietary approaches and eating regimes, including ketogenic and intermittent fasting, are also emerging as beneficial for brain health. This review summarizes the pathophysiology, associated risk factors, and the potential neuroprotective pathways activated by several diets and eating regimes that have shown promising results in promoting brain health and preventing dementia.

## 1. Introduction

### 1.1. Current state of Alzheimer’s dementia research

With age being a prime risk factor for dementia and the rising life expectancy, the global burden of this devastating condition is expected to increase exponentially. According to the Global Burden of Disease (GBD) 2019 study, the disability-adjusted life-years (DALYs) of Alzheimer’s disease (AD) and other dementias is 4.3% of total DALYs (2.02–9.7%) globally and 5.3% of total DALYs (2.66–10.86%) in the United States for individuals 70 years old (Institute for Health Metrics and Evaluation GBD2019).^1^ The number of individuals with dementia is expected to increase from 57.4 (95% CI 50.4–65.1) million cases globally to 152.8 (130.8–175.9) million cases from 2019 to 2050 (1). AD is the most common type of dementia, accounting for at least two-thirds of cases of dementia in patients ages 65 and older (2). Thus, AD and other dementias are projected to be significant health burdens worldwide.

According to the World Health Organization (WHO), AD is a progressive disease divided into three clinical stages. The first stage is mild cognitive impairment (MCI), an intermediate stage between normal cognitive functioning and frank AD dementia. MCI affects individuals’ memory with or without affecting their daily lives (3). Examples of MCI include losing personal belongings and forgetting to attend appointments. MCI may persist throughout life or progress to mild, moderate, and advanced AD forms. In other words, MCI can be considered a precursor of AD. Signs of advanced AD include forgetting long-term memories and loved ones and impairments in completing daily activities such as feeding, toileting, and dressing.

Currently, there are no cures for AD. Several drugs have been investigated as neuroprotective agents for AD. For example, cholinesterase inhibitors, which increase acetylcholine availability, may reduce the progression of cognitive decline; however, there is no substantial evidence that these treatments are neuroprotective or slow the course of the disease (4, 5). Another studied drug, memantine, is an uncompetitive antagonist of glutamate N-methyl-D-aspartate (NMDA) receptors, which plays a role in learning and memory (6, 7). Excessive NMDA stimulation may lead to neurotoxicity (8). Memantine may block the pathological stimulation of NMDA receptors. A literature review, however, suggests that the effects of memantine may be minor and are often not clinically significant (9). Another line of drugs, monoamine oxidase (MAO) inhibitors, can potentially deplete the production of neurotoxic compounds (10). Recently, aducanumab and lecanemab, antibodies directed against amyloid beta (Aβ), the main component of amyloid plaques, have received much attention based on promising results in a meta-analysis of findings from several clinical trials (11). Nevertheless, in a recent lecanemab trial, the clinical benefit was moderate and associated with significant adverse events (12, 13). Certain cholesterol transporters have also been linked to amyloid transport into the brain; therefore, these pathways are also considered potential therapeutic targets (10). Strikingly, as of January 2022, there were 143 potential therapeutic agents in the AD drug development pipeline (14). Unfortunately, most clinical trials have proven futile, and some drugs only alleviate symptoms. Because of the lack of treatment to delay the onset and slow the progress of AD, it is essential to investigate lifestyle changes that may reduce the risk and/or change the course of the disease.

**Figure 1:**
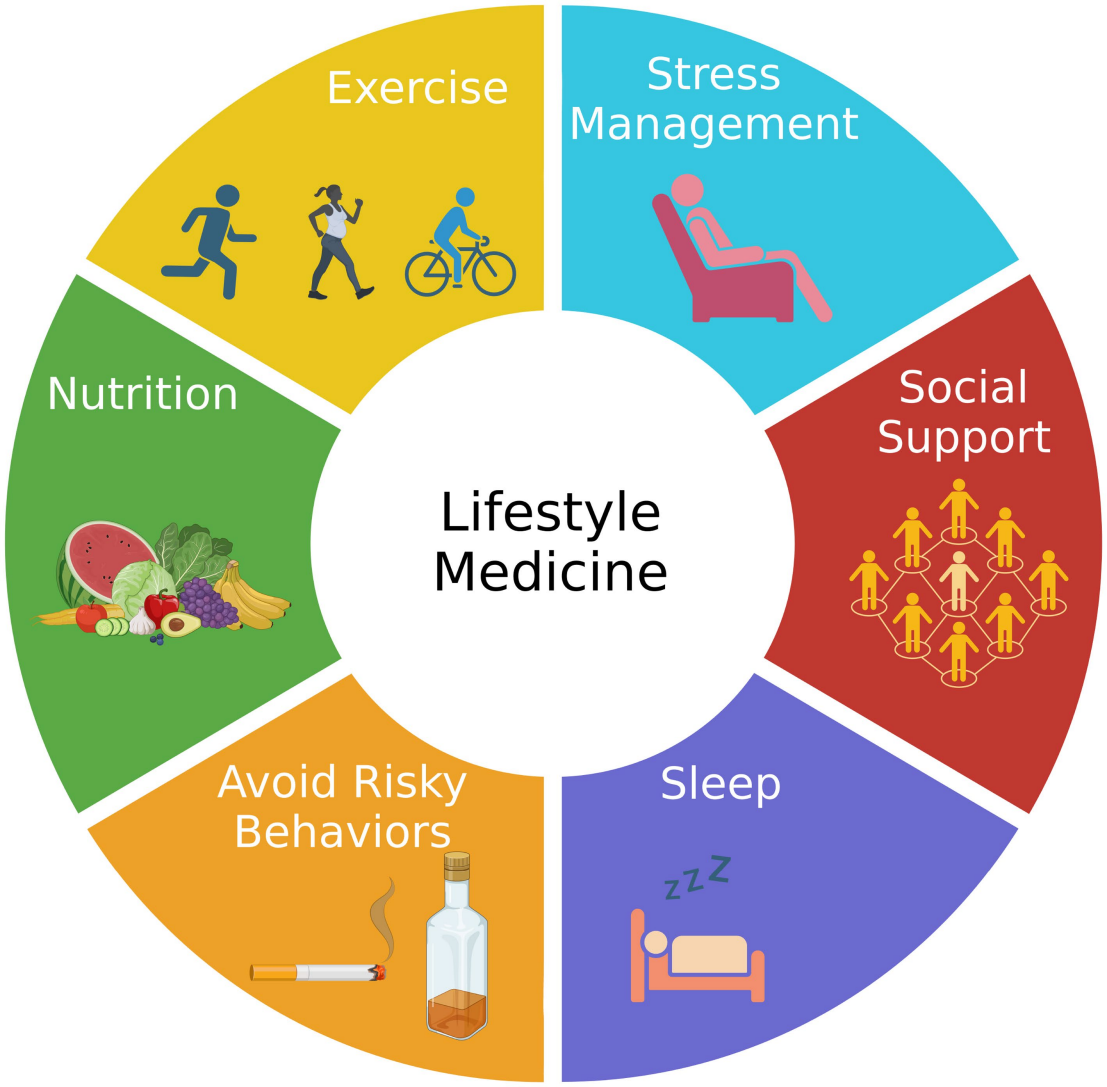
The components of lifestyle medicine. Lifestyle medicine focuses on six pillars of a healthy lifestyle: nutrition, exercise, stress management, social support, sleep, and avoiding risky behaviors, such as smoking tobacco. Adherence to the six pillars is expected to reduce the risk of Alzheimer’s disease.

AD is a highly complex disease comprising multiple genetic and environmental factors, some of which are modifiable. Genetically, there are monogenic and polygenic forms of the disease, with the latter accounting for more than 95% of the cases (15). In other words, most AD cases are not explained by a single genetic cause but are rather influenced by multiple genes in combination with lifestyle and environmental factors. Approximately one-third of AD cases could be related to low educational levels, smoking, alcohol use, depression, diabetes, hypertension, obesity, and physical inactivity (16). Recent advances in network medicine have revealed common molecular and pathophysiological mechanisms shared between AD and other comorbid diseases (17). A hypothesis for the development of AD has been proposed based on genomics (GWAS, whole-genome/exome sequencing, targeted gene sequencing, and functional genomics), transcriptomics (microarrays and RNA-seq), radiomics (brain imaging), pharmacogenomics (drug-target network and drug-gene signatures), and interactomics (protein–protein interactome) that identifies six endophenotypes, including amyloidosis, tauopathy, neuroinflammation, mitochondrial dysfunction, vascular dysfunction, and lysosomal dysfunction (18). The risk for and progression rate of most, and perhaps all, of these AD endophenotypes should respond positively to healthy habits associated with lifestyle medicine, including eating well, being physically active, getting enough good quality sleep, being socially engaged, avoiding exposure to air pollution and second-hand tobacco smoke, quitting smoking, and reducing alcohol drinking (19, 20). A summary of the six pillars of lifestyle medicine is presented in Figure 1.

### 1.2. Diabetes, cardiovascular disease, and Alzheimer’s disease

AD is associated with a wide range of comorbidities (17). T2D and cardiovascular disease are among the most common comorbidities with the most negative impact in AD patients. T2D is the most prevalent metabolic disorder, affecting an estimated 462 million individuals globally (21). AD and T2D share common risk factors, including but not limited to obesity, physical inactivity, depression, family history of diabetes, heart disease, hypertension, or stroke.

In both men and women, having T2D results in approximately a 60% higher risk of developing dementia than those without T2D (22). For this reason, AD is often referred to as type 3 diabetes. Insulin resistance negatively impacts cognition (23). Excess insulin leads to the availability of glucose and fats, which may increase reactive oxygen species that can negatively affect brain health (24). Healthcare providers should therefore assess the risk for MCI and AD in patients with T2D, cardiovascular disease, renal disease, and other complications. For instance, the Mini-Mental Status Exam (MMSE) and the Montreal Cognitive Assessment (MoCA) provide valuable information about the patient’s cognitive status that the physician may use to guide them in delivering specific lifestyle changes that the patient might implement. One crucial modifiable risk factor for AD is a healthy eating pattern. Evidence from epidemiological studies suggests that certain nutrients that reduce the risk of T2D may have protective effects against AD (25). For example, adherence to a Mediterranean diet, including core foods such as fish, olive oil, fruit, and green leafy vegetables, may reduce the risk of AD and cognitive decline (26).

Although the molecular mechanisms that link T2D and MCI with AD are not fully understood, several studies have revealed shared pathways and characteristics between the two diseases. For example, amyloid and tau proteins are common molecular pathological features in AD and T2D (27). In addition, transcription factors, which switch genes on or off, regulate the expression of genes similarly in AD and T2D (28). Notably, inflammation, insulin and glucose metabolism, and the phosphatidylinositol 3-kinase and protein kinase B/Akt (PI3K-AKT) play a pivotal role in developing T2D and AD (28). Thus, targeting shared pathways between both diseases may lead to novel therapeutic therapies for AD.

Brain degeneration is a pathophysiological change shared between MCI, AD, and T2D (29). Specifically, medial temporal lobe atrophy is a common finding in MCI and AD (30). Neuroimaging studies have reported that diabetes is also associated with smaller total cerebral brain volumes and a decline in executive function in individuals without cerebrovascular disease or dementia (31, 32). In addition, chronic hypovolemia, most likely due to dehydration, is present in diabetes, hypertension, and AD (33). Furthermore, total body water decreases as we age (34). Thus, drinking more water would rehydrate the brain (35). Clinical trials are needed to determine whether drinking enough liquids to stay well hydrated may reduce or prevent brain atrophy.

Increasing evidence also suggests a link between AD and atherosclerosis, the buildup of fats in the arteries, which can result from chronic poor dietary choices. One of the shared genetic risk factors is APOEε4, which confers a modest risk for atherosclerosis/heart disease and the development of AD (36).

In summary, T2D, cardiovascular disease, and AD share several mechanistic molecular pathways that lead to disease. The pathways present potential therapeutic targets that may reduce the risk of these chronic diseases. Nutrient-rich eating patterns are expected to reduce the risk or slow the progression of dementia by reducing the risk of T2D and cardiovascular disease.

## 2. Diets beneficial for patients with Alzheimer’s dementia

**Figure 2:**
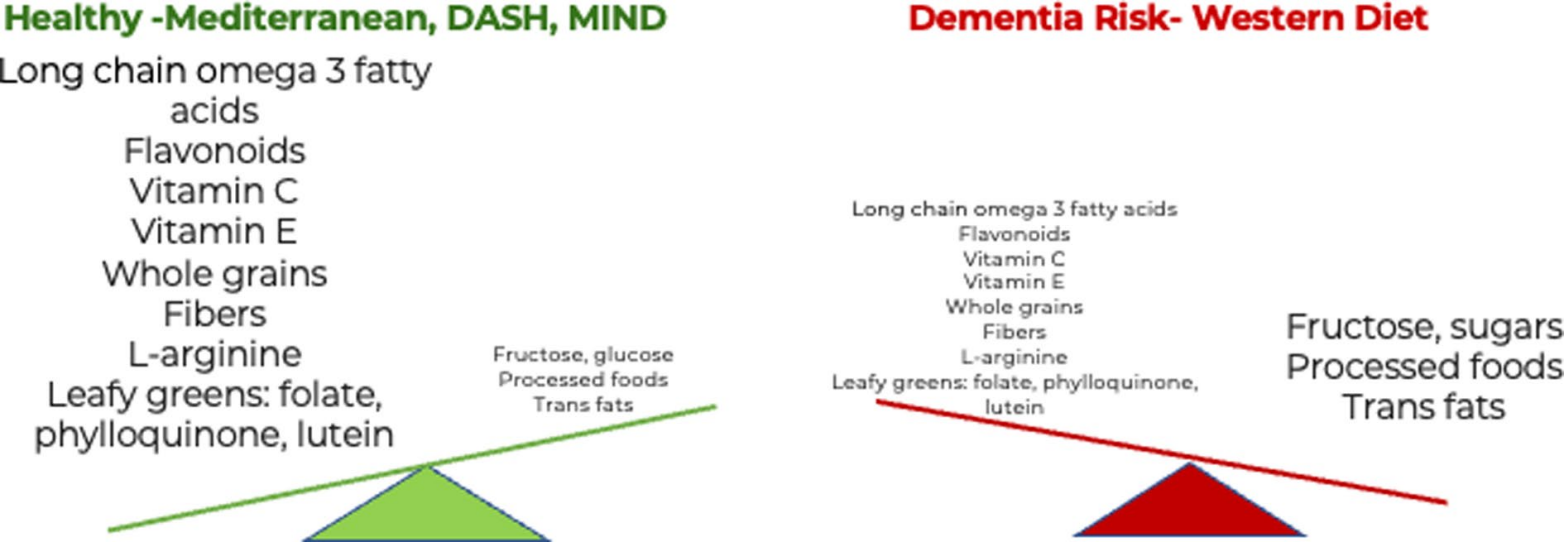
The influence of diet and dietary components in dementia. Eating more nutrient-rich whole foods in a healthy diet reduces the risk of dementia. B. Eating more ultra-processed foods in a Western diet increases the risk of dementia (42).

Studies suggest that metabolism generally slows down during aging and may exacerbate AD (37). Eating a nutrient-rich diet throughout life, beginning at a young age, contributes positively to healthy aging. Multiple challenges to eating a nutritious diet arise with age due to a combination of physiologic, social, environmental, economic, and physical barriers (38). Lifelong adherence to healthy dietary patterns that include whole, unprocessed plant foods, such as those described below, may reduce the risk of dementia later in life (39–41). A summary of healthy eating patterns is presented in Table 1 and is further described below. Selected nutrients within eating patterns are also discussed and depicted in Figure 2.

**Table 1:** Comparison between a Mediterranean, DASH, and MIND Diets.

Amount	Mediterranean	DASH	MIND
Generous (Most meals)	Fruits (unprocessed)	Fruits (unprocessed)	Berries
	Vegetables (unprocessed)	Vegetables (unprocessed)	Leafy Greens
	Whole Grains (unrefined)	Whole Grains	Other vegetables
	Beans		Whole Grains
	Legumes		Beans
	Nuts and Seeds		Legumes
	Extra virgin olive oil		Nuts and Seeds
			Extra virgin olive oil
Moderate (1–3 meals per week)	Fish (not fried)	Fish	Fish (not fried)
	Poultry (not fried)	Poultry	Poultry (not fried)
	Low-fat dairy products	Low-fat dairy products	Alcohol
	Red wine	Nuts, seeds, dry beans, peas	
		Vegetable Oils	
Limit	Sweets	Refined sugar	Pastries and sweets
	Fried foods	Processed foods	Processed foods
	Red Meat	Full-fat dairy products	Red meat
	Processed meat	Saturated fats	Fried foods
	Refined oils	Trans fats	Cheese
	Butter/cream	Sodium	

The Mediterranean, DASH, and MIND Diets were compared to identify shared components. A nutrient-rich diet that includes these ingredients is expected to reduce the risk of AD.

### 2.1. Mediterranean diet

The Mediterranean diet is an eating pattern influenced by the cuisines surrounding the Mediterranean Sea. Key features of the diet include ample plant-based, unprocessed foods, including whole grains, fresh vegetables, beans, legumes, seeds, extra virgin olive oil, nuts, and fruits (41, 43). Foods such as fatty fish, moderate amounts of lean poultry, low-fat dairy products, and red wine are often part of a Mediterranean diet. The Mediterranean diet limits butter/cream, red meats, processed foods, sweets, refined oils, and fried foods (44). More than an eating pattern, the Mediterranean diet can be considered a lifestyle. Individuals in the Mediterranean region often stay active, socialize, reduce stress, and get adequate sleep (45, 46), all critical determinants in the development of dementia.

Adherence to the Mediterranean diet has been shown to improve health outcomes across many diseases, including cognitive decline and neurodegeneration (47, 48). Evidence from molecular and epidemiological studies supports a neuroprotective effect of the Mediterranean diet in cognitive decline and dementia. For instance, a comparison of gene expression data from the blood of individuals with dementia and those who followed the Mediterranean diet were inversely correlated and showed that consuming omega-3 fatty acids from foods such as nuts, olive oil, and fish may help reduce the risk of dementia (42). Indeed, foods common to a Mediterranean-style diet have anti-inflammatory, antioxidant, and neuroprotective properties (49). For example, consuming long-chain omega-3 fatty acids from fish and polyphenols from fruit, red wine, and tea have positively affected brain health and cognition in older adults (50). Recently, longitudinal analysis derived from individuals without dementia in the Alzheimer’s Disease Neuroimaging Initiative (ADNI) revealed that long-term use of omega-3 fatty acid supplements exhibited a 64% reduced risk of AD (51). The same study suggested that dietary omega 3, especially docosahexaenoic acid (DHA), could reduce the risk of all-cause dementia and cognitive decline by 20%.

Several clinical trials have found positive health outcomes of the Mediterranean diet on brain health in several populations. For instance, a randomized controlled trial (RCT), the PREDIMED-NAVARRA study in Spain of 522 individuals at high vascular risk, showed that participants allocated to the Mediterranean diet displayed higher MMSE and Clock Drawing Test (CDT) scores compared to those on a low-fat diet after 6.5 years of nutritional intervention (52). Similarly, a trial using a randomized subgroup of 285 participants from the same population cohort showed that a Mediterranean diet supplemented with extra virgin olive oil improved cognitive function compared to a low-fat diet after 6.5 years of nutritional intervention (53). These results were obtained after adjustment for risk factors such as sex, age, education, physical activity, body mass index, diabetes, hyperlipidemia, smoking, and hypertension. A prospective French cohort study of 1,410 adults (≥65 years) found that higher adherence to a Mediterranean diet was associated with slower MMSE cognitive decline but not with risk for incident dementia over a 5-year period (54). Furthermore, a systematic review and meta-analysis of fifteen cohort studies and two RCTs revealed significant positive associations between the Mediterranean diet and episodic memory and global cognition but not working or semantic memory (55).

In contrast, some RCTs have not found beneficial effects of the Mediterranean diet on cognitive functions. For instance, the MedLey study conducted in Australia, which allocated participants to a Mediterranean diet, did not show improvement in cognition among healthy older adults (56). This cohort included 137 men and women randomly assigned to either a Mediterranean diet or control diet for 6 months. In this regard, the duration of this trial was shorter compared to previous trials reporting positive outcomes. A systematic review and meta-analysis of 5 RCTs determined that the effects of the Mediterranean diet on cognition and brain morphology and function were mainly non-significant with small effect sizes (57), except the significant associations of 3 composite cognitive scores in memory, frontal, and global function obtained from PREDIMED trial (52, 53). Nevertheless, this study notes that the prescribed Mediterranean diets varied considerably among the studies, possibly introducing bias in the analysis.

In addition to cognitive functions, several groups have investigated the effects of the Mediterranean diet on brain structure and neuropathological features of AD. For example, a higher Mediterranean diet adherence score was associated with lower amyloid beta accumulation in 77 cognitively normal subjects from the Australian Imaging Biomarkers and Lifestyle Study of Aging after a 3 years period (58). The group of cognitively normal subjects was described as Aβ accumulators suggesting these patients were likely to develop AD. Other investigations have explored the relationship between the Mediterranean diet and structural neuroimaging markers. For example, a cross-sectional study of 52 cognitively normal individuals reported that those with a higher adherence to a Mediterranean diet showed a greater thickness in AD-vulnerable brain regions (59). Conversely, individuals with a lower adherence to a Mediterranean diet displayed cortical thinning in the same brain regions as clinical AD patients. Another cross-sectional study on 674 older adults without dementia showed that higher adherence to a Mediterranean diet was associated with less brain atrophy in areas commonly affected in AD, including the cingulate cortex, parietal lobe, temporal lobe, and hippocampus (60). The authors of this study suggested that higher fish and lower meat intake might be critical nutrients for preserving brain structure. In contrast, some studies reported no significant associations between adherence to the Mediterranean diet and gray matter volumes at baseline or longitudinally (61–63). One of these studies, however, found a negative association between self-reported intake of meat and total brain volume (63).

Despite the inconsistency among the studies on cognition, several studies have shown that adherence to a Mediterranean diet may prevent the development of cardiovascular disease, diabetes, and depression, which are implicated in dementia. Several systematic reviews and meta-analyses have revealed that a Mediterranean diet reduces the risk of diabetes (64–66). Furthermore, cardiovascular disease has been associated with an increased risk of dementia and AD (67, 68). In this regard, results from the PREDIMED study, a multicenter trial in Spain comprising 7,447 participants, revealed that individuals at high cardiovascular risk assigned to a Mediterranean diet had a lower incidence of major cardiovascular events (69). A meta-analysis of 22 studies found that high adherence to a Mediterranean diet was protective against ischemic stroke, depression, MCI, dementia, and particularly AD (70). Similarly, several studies have found a reduced risk of depression and the development of depressive symptoms in subjects adhering to a Mediterranean diet (71, 72).

These studies highlight the protective role of a Mediterranean diet on brain health and the potential prevention of chronic diseases such as dementia. The neuroprotective potential afforded by the Mediterranean diet is further reinforced by studies showing a reduced risk of cardiovascular disease, diabetes, and depression, known risk factors for dementia.

### 2.2. DASH Dietary Approaches to Stop Hypertension (DASH) diet

The Dietary Approaches to Stop Hypertension (DASH) diet is a healthy eating pattern emphasizing the consumption of whole grains, fruits, and vegetables. DASH mainly focuses on dietary choices to promote heart health and prevent hypertension (39). The DASH diet also recommends limiting fats, particularly saturated and trans fats, and processed foods, which are the leading contributor to dietary sodium, as well as sweets and sugar-sweetened beverages that contribute calories but otherwise have little nutritional value.^2^ Further details regarding the DASH-recommended eating pattern are highlighted in Table 1.

Cross-sectional and longitudinal studies have shown promising results on DASH diet interventions and cognitive decline. A cross-sectional study including 164 adults without dementia showed that a DASH diet was associated with improved verbal memory but not executive function or visual memory (73). Additionally, a prospective cohort study comprising 824 subjects enrolled in the Memory and Aging Project (MAP) showed that adherence to a DASH diet resulted in a slower rate of decline in global cognition in older adults (74). The MAP study is an ongoing cohort of cognitively normal adults living in Chicago retirement communities (75). MAP participants agree to cognitive and dietary assessments. Adherence to DASH dietary recommendations (defined as nine or more DASH-associated foods or nutrients) was associated with better than average cognitive function, regardless of APOEε4 gene status in 16,144 older women enrolled in the Nurse’s Health Study (76). Strict adherence to DASH and Mediterranean diets may be needed to produce notable effects (40).

Though promising results have been reported, some studies have not found a protective effect of the DASH diet on cognition or dementia. The longitudinal Women’s Health Initiative Memory Study comprising 6,425 cognitively intact post-menopausal women determined that a DASH dietary pattern was not associated with cognitive decline (77). Another study revealed that a DASH diet was neither associated with MMSE nor a cognitive decline in Swedish older adults (78). More extensive longitudinal trials are needed to determine the effects of the DASH diet on brain health.

### 2.3. Mediterranean-DASH Intervention for Neurodegenerative Delay (MIND) diet

The Mediterranean-DASH Intervention for Neurodegenerative Delay (MIND) diet was developed specifically for its neuroprotective effects by a team at Rush Medical Center and is a dietary pattern that combines Mediterranean and DASH diets (40). Like Mediterranean and DASH diets, the MIND diet encourages a predominantly plant-based nutritional pattern consisting of leafy green vegetables, other vegetables, berries, nuts, whole grains, and legumes and limiting amounts of processed sweets, fried foods, and cheese. Compared to the Mediterranean and DASH diets, the MIND diet plan specifically emphasizes berry consumption and the integration of leafy green vegetables into most meals (40).

Research findings suggest that the MIND diet might protect against AD development independent of other healthy lifestyle behaviors and cardiovascular-related conditions (40). Cross-sectional and prospective studies on the MIND diet have shown promising findings on brain health in several populations. Greater adherence to the MIND and Mediterranean diets is associated with improved cognitive function and lower risk of cognitive impairment in older adults participating in The Health and Retirement Study (79). Longitudinal studies have supported these findings. For instance, MIND but not the Mediterranean diet, was associated with reduced odds of cognitive impairment in a 12-year Australian longitudinal cohort (80). Another longitudinal study showed that a higher MIND diet score was associated with reduced cognitive decline in a Swedish population (78). Even some adherence to the MIND diet may have substantial benefits for AD prevention. For example, in one trial, higher strawberry intake was associated with a reduced risk of AD (81).

Similar to the other dietary patterns, several studies on the MIND diet have not yielded positive outcomes on cognition and dementia. For example, results from the US Nurses’ Health Study showed that the MIND diet was not significantly associated with cognitive decline or verbal memory in older women (82). Nevertheless, the same study suggested that long-term adherence to the MIND diet was associated with improved verbal memory later in life. A recent study within the UK Biobank showed that following the MIND diet was not associated with enhanced cognitive test scores (83). The MIND score was derived from 24 h diet recall questionnaires for 120, 661 individuals who completed at least one self-administered cognitive function test. Notwithstanding, there were some noteworthy differences from the original MIND score with regards to some components, including olive oil, vegetables, and pastries and sweets. For example, the questionnaire used did not ask about primary oil for cooking, and participants were assigned a point if they reported the use of fat/oil in cooking. It is also important to note that more evidence-based research is needed over an extended period to support the MIND diet’s benefits for reducing the risk of dementia.

### 2.4. Ketogenic diet

Ketogenic (“keto”) diets, which are classified as low carbohydrate and high fat (in the form of healthy fats, such as nuts, olive oil, and avocado), have also been widely studied. The diet involves the restriction of carbohydrates to the point where the body produces ketones, which can be an alternative, beneficial fuel for the brain (84). A ketogenic diet is also recommended for various medical conditions, including liver disease, insulin resistance, and other neurological disorders (85, 86).

Glucose is the primary source of energy for the brain. With aging and the development of neurodegenerative diseases, the brain becomes less efficient at using glucose (87). For instance, AD has been linked to insulin resistance and the loss of glucose transporters, which further disturbs brain functioning (86). Ketones may provide an alternative energy source for the brain with prominent insulin resistance. Studies have shown that ketogenic diets may decrease neuroinflammation (88), reduce the production of free radicals (84), and reduce amyloid plaque formation (88). Adhering to a low-carbohydrate diet has also been linked to improved memory and cognitive function in older adults (89). Adherence to a ketogenic diet improved working memory, visual attention, and task switching in non-demented adults over 60 years old (90). The ketogenic diet included an oral intake of a ketogenic meal (Ketonformula) containing 20 g of medium-chain triglycerides. Similarly, using the same ketogenic formula daily for up to 12 weeks showed positive effects on verbal memory and processing speed in Japanese patients with mild to moderate AD (91). A recent study suggested that a high-fat modified ketogenic diet may benefit prediabetic patients with MCI by modulating the gut microbiome, mainly through regulating GABA-producing microbes and gut transit time (92). This study highlights the importance of early intervention, specifically in prediabetic patients with MCI, for successful therapeutic outcomes. In addition to its benefits on cognition, AD, and prediabetes, preclinical models have suggested that a ketogenic diet may be helpful for anxiety and depression. It has been posited that the production of ketone bodies derived from a ketogenic diet may act as glutamate inhibitors in the NMDA extrasynaptic receptor, decreasing inflammation and oxidative stress (93). Nevertheless, more evidence-based research is needed before determining whether adherence to ketogenic diets reduces the risk of dementia (85, 94) and psychiatric diseases (93, 95).

## 3. Common nutrients in healthy and plant-based diets

Understanding the common food sources and nutrients in healthy dietary patterns helps formulate recommendations that promote beneficial lifestyle changes. Unsaturated fatty acids, including monounsaturated fatty acids (MUFAs) and polyunsaturated fatty acids (PUFAs), have been studied for their potential anti-inflammatory and neuroprotective effects (96). Olive oil, the primary source of fat in the Mediterranean and MIND diets, has widely accepted health benefits. Olive oil is rich in phenolic compounds and fatty acids such as oleic acid, the primary MUFA in olive oil (97, 98). Supplementing lab mice with oleic acid and decreasing cholesterol intake has been shown to reduce amyloid plaques in the brain, which indicates it may have preventative effects (99). In the European Prospective Investigation into Cancer and Nutrition (EPIC-Spain) study, mortality rates were measured 13 years later (100). Researchers found that for each increase in olive oil consumption by 10 g · 2000 kcal^−1^ · d^−1^, there was a 7% decreased risk of overall mortality and a 13% reduced risk of cardiac-related mortality (100).

Evidence suggests that nuts, such as walnuts, almonds, and hazelnuts, contain various bioactive components that may support brain function (101). Most nuts are good sources of MUFAs and other nutrients such as fibers, vitamin E, and L-arginine (42). Walnuts are specifically rich in long-chain omega-3 fatty acids. The walnut extract has been shown to inhibit the formation and persistence of amyloid proteins; clinical trials in adults have associated walnut consumption with improved memory and cognitive performance reviewed by (102). The health benefits of omega-3 fatty acids have been extensively documented in several populations. Results from The Framingham Heart Study, which included 2,183 dementia and stroke-free individuals, showed that a higher intake of omega 3 was associated with larger hippocampal volumes and better abstract reasoning (103). A systematic review of 12 clinical studies indicated that a higher omega-3 fatty acids consumption is associated with larger volumes in the hippocampus, total grey matter, total brain volume, and lower white matter lesions (104). Interestingly, several studies have reported an interaction between vitamin B and omega-3 fatty acids suggesting that omega-3 fatty acids influence the efficacy of vitamin B in slowing cognitive decline (105). One study showed that the beneficial effect of vitamin B on brain atrophy was only observed in individuals with high plasma omega-3 fatty acids (106). Therefore, a careful assessment of interactions among different nutrients and their synergistic effects needs to be considered in nutritional interventions.

Consuming various unprocessed fruits and vegetables, abundant in naturally occurring vitamins and minerals, is a healthy dietary practice. Eating at least three servings of vegetables and two servings of fruits daily, as the WHO recommended, may aid in keeping us well-hydrated and preventing dementia later in life. A six-year, longitudinal study in Hong Kong aimed to assess fruit and vegetable consumption and its link to cognitive function in 17,700 older adults without dementia. It was determined that dementia risk was lower in those who consumed fruits and vegetables daily (107). Consumption of green leafy vegetables provides the body with fiber, folate, phylloquinone, and lutein and was associated with slower cognitive decline in an older US population (108). In addition, fruits and vegetables contain abundant water (109).

Red wine, another critical component of the Mediterranean diet, is rich in phenolic compounds. One of these phenolic compounds, resveratrol, has been shown to have antioxidant effects that may prevent Aβ toxicity (110). A follow-up of 5,033 subjects from the longitudinal Tromsø study conducted in Norway found that light-to-moderate wine consumption was associated with better performance on cognitive tests 7 years later (111). Another study looked at resveratrol specifically and found that it was linked to the regulation of neuroinflammation (112). Resveratrol derivatives have also been implied as potential therapeutic targets in AD (113). Due to the risk factors between alcohol consumption and other health conditions, red wine should be consumed in moderation because the beneficial effects have been linked to resveratrol, not ethanol (alcohol) (114). Averaging more than 12 grams of alcohol daily may increase dementia risk (115).

It has been shown that plant-based/vegan diets can also benefit insulin sensitivity and cognition (116). However, besides emphasizing a dietary pattern rich in unprocessed plant foods, fish is one animal-based food recommended by the Mediterranean and MIND diets. Fish oils are a predominant dietary source high in omega-3 fatty acids. In subjects with pre-existing heart conditions, fatty fish consumption led to decreased lipids, potential mediators of insulin resistance and inflammation (117). This suggests that fish consumption may reduce the risk of the progression of insulin resistance and T2D (118). Another analysis showed an association between increased fish intake and decreased cognitive decline, notably in episodic memory (119).

## 4. Other nutritional sources with neuroprotective potential

### 4.1. Spices

Spices are key components of many different cuisines worldwide. Cinnamon, curcumin-turmeric, and capsaicin from chili peppers have been studied for their role in reducing inflammation, improving blood glucose, boosting memory and cognition, and potentially preventing dementia. For example, cinnamon has been widely documented to potentiate insulin activity, lower blood glucose and cholesterol, and reduce inflammation, thus representing a natural adjunct treatment for T2D and neurodegenerative diseases (120, 121). Cellular and animal studies have reported that cinnamon may be neuroprotective against AD. For example, cinnamon extracts have been shown to inhibit tau aggregation and the buildup of Aβ plaques in cellular and animal models (122, 123). A systematic review including 40 preclinical and clinical studies reported that cinnamon might be a helpful adjuvant treatment for cognitive decline (124). However, it is noteworthy that most research is based on chemically extracted components from cinnamon, not the natural spice itself.

Like cinnamon, turmeric has anti-inflammatory, antioxidant, and neuroprotective properties. Specifically, the beneficial effects of turmeric on brain health are attributed to the curcumin component of turmeric. Numerous *in vitro* and *in vivo* studies have shown that curcumin and curcuminoids reduce the levels of Aβ, suppress beta-secretase 1 (BACE1) activity, and inhibit Aβ aggregation (125–127). Curcumin and its derivatives have been shown to modulate Aβ production, aggregation, and clearance *via* the regulation of the Wnt/β-catenin, autophagy, and the unfolded protein response pathways (127, 128). Despite these positive findings, a randomized double-blinded clinical trial showed no benefits from a curcumin formulation on cognition in older adults (129).

Capsaicin, the main chemical component in chili peppers, provides a spicy flavor to many different cuisines worldwide. It has been implicated in various biological processes, including obesity, cerebrovascular function, cognition, neuroprotection, and the regulation of the gut microbiome (130–133). For example, capsaicin-mediated activation of the TRPV1 channel reversed the impairments of long-term hippocampal potentiation and spatial learning and memory in APP23/PS45 double-transgenic AD mice (134). Similarly, activating TRPV1 by capsaicin rescued metabolic defects of microglia, decreased amyloid pathology, and reversed memory deficits in a mouse model of AD (135). In a population study in China, chili pepper consumption, represented as total capsaicin scores, was positively correlated with MMSE scores and inversely associated with serum Aβ40 and total serum Aβ levels (136). Nevertheless, the evidence on the neuroprotective potential is inconclusive. In this regard, in a Chinese longitudinal cohort, chili intake was associated with cognitive decline, particularly in those individuals with low BMI (137). The same group reported an inverse association between chili consumption and obesity and hypertension (138, 139). Note that this study analyzed chili consumption, not capsaicin alone. Indeed, most of the spices are commercially available as powders, and most of the research results are not derived from the spices in their naturally occurring form. In addition, it remains unclear the potential interactions between chili pepper, obesity, hypertension, and cognitive decline. Therefore, more longitudinal studies considering different herbs/spices formulations (i.e., chemical extract vs. naturally occurring form), genetics, obesity, hypertension, and other environmental factors are critical to determining its therapeutic potential.

### 4.2. Cocoa

Several groups have explored the potential effects of cocoa and cocoa-derived products on cognitive functions. Cocoa has been recognized as a rich source of flavonoids, particularly in the form of epicatechin and catechin, with multiple health benefits, including cardioprotection, neuroprotection, and neuromodulation reviewed by (140). The rationale for the neuroprotective effects of cocoa flavonoids stems from their ability to cross the blood–brain barrier and exert their antioxidant effects in brain regions crucial for learning and memory, such as the hippocampus, cerebral cortex, and striatum (140, 141). In addition, preclinical models indicate that individual flavonoid molecules reduce activated microglia and inflammation and inactivate inflammasome-related transcription factors (142). Randomized clinical trials demonstrated that cocoa flavanol intake for 8 weeks improved cognitive function and decreased insulin resistance, blood pressure, and lipid peroxidation in subjects with MCI and cognitively intact older adults (143, 144). The authors suggested that the protective effects of cocoa flavanols were partly mediated through improvement in insulin sensitivity. The evidence from clinical trials is limited. More extensive prospective trials using cocoa-derived products are needed to verify these findings.

### 4.3. Lion’s mane mushroom

Growing evidence indicates that some mushrooms provide multiple health benefits, including anti-inflammatory, anti-depressive, antidiabetic, and neuroprotective properties (145). Hericium erinaceus, best known as Lion’s mane mushroom, is one of the most studied. *In vitro* and *in vivo* studies have shown that this mushroom possess antioxidative, anti-inflammatory, and neuroprotective properties beneficial for the treatment of neurodegenerative diseases and depression (145–147). For example, a 30-day oral intake of Hericium erinaceus attenuated cerebral amyloid beta plaque burden in the APPswe/PS1dE9 transgenic mouse model of AD (148). In addition, components of Hericium erinaceus mycelium ameliorated amyloid beta plaque, reduced the activation of glial cells, raised the level of insulin-degrading enzyme, and promoted hippocampal neurogenesis in APPswe/PS1dE9 transgenic mouse model of AD (149). The neuroprotective effects of Hericium erinaceus constituents promoting neuronal survival and neurogenesis may be mediated *via* the TrkA/Erk1/2 pathways (150).

Despite these findings, very few human studies have investigated the effects of Lion’s mane on memory and cognition. For example, a double-blind placebo-controlled trial reported that intake of 250 mg tablets containing Hericium erinaceus for 16 weeks improved mild cognitive impairment in Japanese men 50–80 years old (151). Likewise, another double-blind placebo-controlled trial showed that consumption of Hericium erinaceus for 12 weeks improved cognitive functions (152). In contrast, a single-blind placebo longitudinal trial showed that intake of 10 g of Hercium erinaceus for four weeks had no impact on metabolic markers or cognition in younger adults (153). Given the limited number of human studies, larger prospective clinical trials are needed to verify the neuroprotective effect of Hericium erinaceus in patients at risk for cognitive decline and dementia.

### 4.4. Lithium

Growing evidence indicates that lithium has neuroprotective properties. Lithium salts are well established as a mood stabilizer in treating neuropsychiatric conditions, including mania, bipolar disorder, and treatment-resistant major depression. Preclinical and molecular studies have shown that lithium confers neuroprotection *via* the inhibition of the enzyme glycogen synthase kinase 3β (GSK-3β) reviewed in (154) and stimulation of BDNF and VGF growth factors (155, 156). Lithium treatment reduces p-tau and neurofibrillary tangles formation in animal models of tauopathies and AD (157–159). In addition, lithium treatment stabilized neuronal signaling alterations, including aberrant Ca^2+^signaling, restored neuronal nitric oxide synthase (nNOS) and p-tau levels, and enhanced short-term plasticity in the hippocampus of 3xTg-AD mice (160). Nevertheless, the evidence obtained in clinical trials is mixed. For example, an RCT showed that a lithium microdose of 300 μg prevented cognitive decline in AD subjects 3 months after beginning the treatment (161). Similarly, a small double-blind, placebo-controlled RCT comprising 45 participants showed that lithium treatment (0.25–0.5 mmol/l) was associated with decreased CSF concentrations of p-tau and improved cognitive performance in individuals with amnestic MCI (162). In contrast, a multi-center RCT found no evidence of lithium neuroprotection in mild AD patients after 10 weeks of treatment (163). Although the evidence is inconclusive, future larger longitudinal studies investigating the therapeutic effects of lithium in early-stage MCI patients are warranted. In addition, since microdoses of lithium have shown promise, investigating lithium-containing (such as tomatoes, cabbage, nutmeg, coriander seed, and cumin) and lithium-fortified foods should be explored in the context of neuroprotection (71). It is also noteworthy to mention lithium’s reported adverse effects and toxicity burden on several organs, including the kidneys, thyroid gland, and parathyroid glands (164).

In summary, despite the evidence supporting the beneficial effects of these nutrients, more research is needed on larger groups of mixed populations over extended periods to determine the benefits of reducing the risk of cognitive impairment. It is clear, however, that an overall healthy eating pattern provides the most impactful long-term health benefits for various diet-related conditions, including reducing the risk of dementia.

## 5. The Western diet and dietary components associated with increased dementia risk

While it is informative to know which foods and nutrients a person should eat to boost neuroprotection, it is also critical to identify foods that a person should limit, such as certain animal products and ultra-processed foods that contain added salt, fat, and sugars and are linked to dementia risk (Figure 2). Several foods and nutrients are known to increase the risk of dementia. A recent systematic review indicated that adherence to the Western diet increases the risk of developing AD, whereas the Mediterranean diet, ketogenic diet, omega-3 supplements, and probiotics are neuroprotective (165). Pro-inflammatory foods are abundant in the Western diet and include ultra-processed foods (UPFs), which are often low in naturally occurring vitamins, minerals, and fiber and instead contain refined grains, added saturated fat and highly processed fats, sugars, red meats, dairy, and fried foods. UPFs, as classified by NOVA, a food classification system based on the nature, extent, and purpose of industrial food processing, contribute more than 60% of energy in US diets (166). Processing alters naturally occurring healthy fats that may be beneficial for maintaining cognition with advancing age. The processing of foods depletes naturally occurring essential nutrients and often adds fat, sugar, and sodium, which contribute to dementia risk (167). Saturated fatty acids have been found to lead to increased production of amyloid proteins (168) and can more than double the risk of AD development (169). Consumption of trans fat, found in processed and fast food, might also increase the risk of AD (170). Processed and refined oils may lose essential antioxidant and anti-inflammatory components, supporting the recommendation to choose pure extra virgin olive oil (171).

In addition, consuming added sugars common in UPFs and notably in sugar-sweetened beverages has also been linked to cognitive decline (172). Recently, it was proposed that diets rich in glucose, fructose, high-fructose corn syrup, high glycemic carbohydrates, and salt lead to brain atrophy, neuron loss, and, ultimately, AD (173). These components are frequently found in a Western diet (Figure 2). Usually, 1–2% of dietary fructose reaches the brain (174). Raising blood glucose levels, dehydration, or the intake of salty foods increases brain fructose levels (175, 176). Dietary fructose also increases fructose production in the brain, potentially by increasing uric acid levels (177, 178). There is also evidence that fructose production and metabolism are elevated in the regions of the brain affected by AD in patients, including the hippocampus, entorhinal cortex, middle temporal gyrus, cingulate cortex, sensory and motor cortex, and cerebellum (179). With regards to salt, more than 70% of the sodium in the US diet comes from processed and restaurant foods, according to the American Heart Association (180). High salt intake can lead to hypertension and is associated with neurodegeneration. Salt has been shown to contribute to blood vessel dysfunction and has a negative effect on cognitive function (181). Based on these studies, reducing the intake of simple sugars and salt and maintaining appropriate hydration levels is expected to reduce the risk of AD.

In summary, a Western-style diet high in UPFs contains more pro-inflammatory foods and dietary components contributing to dementia. In contrast, a dietary pattern emphasizing whole, unprocessed, anti-inflammatory foods may prevent cognitive decline (182). This further emphasizes a whole food, plant-predominant diet, and similar eating patterns for neuroprotection. Emphasizing a lifelong eating pattern that includes both health-promoting nutrients and food while at the same time minimizing potentially harmful dietary components is the best current guidance.

## 6. Lifestyle factors beneficial for patients with Alzheimer’s dementia

Lifestyle medicine is an approach to medicine that integrates evidence-based lifestyle therapeutic intervention as a primary modality. Clinicians can work with patients to examine and modify lifestyle measures to prevent, treat, and often reverse chronic disease. The pillars of lifestyle medicine, which include good nutrition, regular exercise, stress reduction, sleep optimization, social support, and a reduction in tobacco and alcohol abuse, can effectively be included in the clinical efforts to prevent and treat patients with AD and other forms of dementia with none of the side effects associated with medications (Figure 1).

Additionally, increased physical activity reduces the risk of dementia, whereas a sedentary lifestyle increases AD risk (16, 183). Thus, clinicians should recommend specific activities based on patients’ abilities, accommodations, and access to physical activity. For instance, yoga has been linked to neuroprotection and improved visuospatial functioning in elderly individuals with MCI (184). Social support is also a vital component of a healthy lifestyle. Interestingly, sharing stories about one’s life experiences may also encourage problem-solving, improve mood, and enhance memory in elderly individuals with and without MCI (185).

In addition to the basic tenants of lifestyle medicine, recent studies have shown that additional lifestyle changes may be beneficial for reducing the risk of dementia and AD. In a longitudinal study of 2,315 healthy middle-aged Finnish men, 2–3 weekly sauna bathing sessions reduced the risk of dementia and AD (186). In a small pilot study, elderly individuals who were healthy (*n* = 7) or had MCI (*n* = 14) were trained for intermittent hypoxia-hyperoxic training (IHHT) for a total of 15 sessions over 3 weeks. Each IHHT session comprised four cycles of 5-min hypoxia and 3-min hyperoxia. The results from the study showed that IHHT sessions might improve cognition in individuals with MCI and, therefore, may be beneficial for slowing the development of AD (187). These studies indicate that more research is needed to explore the potential lifestyle changes that may benefit individuals with MCI or at risk for AD.

### 6.1. Intermittent fasting

Unlike the dietary patterns described previously, intermittent fasting is not considered a diet but rather an eating regime that includes frequent periods with little or negligible food amounts within a period sufficient to switch to a metabolic state in which glucose, fat-derived ketone bodies, and free fatty acids are used as the primary energy sources. Different intermittent fasting methods include alternate day fasting (ADF) or complete water-only intake every other day, time-restricted fasting (TRF), where food intake is restricted to 6–12 h per day, and the 5:2 diet, which includes 500–700 calories for 2 days per week (188). The numerous positive effects of intermittent fasting in dementia have been noted, including improved inflammatory response, promotion of neurotransmitter secretion, synaptic plasticity, suppression of vascular inflammation, improved brain insulin resistance, and the promotion of neurogenesis (189). Preclinical and clinical studies have shown numerous benefits of intermittent fasting and its potential to modify or prevent many diseases, including obesity, diabetes, cardiovascular disease, cancer, immune diseases, and neurodegenerative disorders reviewed by (190).

In the context of neurodegeneration, evidence from animal models suggests that ADF can delay the onset and progression of AD and Parkinson’s disease (191, 192). Intermittent fasting has been shown to enhance neuronal stress resistance through multiple pathways, including mitochondrial function, autophagy, neurotrophic factor production, neutralization of free radicals, and protection against oxidative stress (191). Calorie restriction or ADF dietary regimens reduced cognitive decline in a triple transgenic mouse model of AD (3xTgAD) (193). Interestingly, 3xTgAD mice in the 40% calorie restriction but not in the ADF regime exhibited lower levels of Aβ_1-40,_ Aβ_1-42,_ and p-tau in the hippocampus compared to the control group. Similarly, 4 months of protein restriction cycles with supplementation of nonessential amino acids decreased p-tau in the hippocampus and alleviated cognitive impairment in 3xTgAD mice (194). Moreover, 12 months of time-restricted feeding improved cognition and enhanced microbiome diversity suggesting a link between gut flora and brain health (195).

Studies of intermittent fasting in humans have also shown promising results. For example, a clinical study in a small Tunisian cohort who observed Ramadan showed that intermittent fasting improved executive function, attention, inhibition, associative memory, and recognition memory (196). Despite the promising findings, several studies have raised concerns about harmful side effects with extended or too frequent fasting regimes. Gallstone disease and increased risk of mortality from cardiovascular disease are among some of the adverse side effects reported in epidemiological studies (197, 198). Therefore, older adults at high risk of neurodegenerative diseases should undergo a careful physical evaluation by a healthcare professional before establishing a fasting regime.

### 6.2. Individualized lifestyle programs for the management of patients at risk for Alzheimer’s dementia

Approximately 35% of dementia cases may be caused by nine modifiable risk factors, including limited education, hypertension, obesity, diabetes, smoking, physical inactivity, depression, social isolation, and hearing loss (19). Healthy eating patterns and other healthy lifestyle factors may benefit those at risk for dementia and neurocognitive impairment. Specifically, establishing healthy plant-predominant eating patterns emphasizing abundant whole, unprocessed foods that provide essential naturally occurring nutrients while minimizing harmful dietary components is associated with neuroprotection. Evidence supports that healthy nutritional habits, such as Mediterranean, MIND, and ketogenic diets, should be considered in preventative health. Adapting all pillars of lifestyle medicine practices can benefit cognitive health.

Ideally, a personalized non-pharmacologic multi-domain intervention will become standard care for individuals at risk for AD. Several studies currently use lifestyle approaches to target the AD pathological pathways, including glucose hypometabolism, inflammation, oxidative stress, amyloid plaque accumulation, and trophic factors (19, 199, 200). The Finnish Geriatric Intervention Study to Prevent Cognitive Impairment and Disability (FINGER) study is a large longitudinal randomized controlled trial that showed a multi-domain approach that includes nutrition, physical activity, and cognitive training reduces the risk of dementia in the aging population (20). The FINGER model is now being tested globally to determine if it effectively reduces the risk of dementia in diverse populations in the World-Wide FINGERS network (201). In a pilot study, aerobic exercise combined with a DASH diet improved executive function in cognitively impaired individuals (202). Likewise, combining a DASH diet and a behavioral weight management program, including exercise and caloric restriction, improved executive function in sedentary and obese individuals (203). In another study, an intervention that increased cognitive, physical, and social activity over 2 years benefited black individuals with MCI (204). In a recent prospective clinical trial, participants were given individualized, multidomain intervention recommendations guided by clinical and biomarker data. Recommendations included education and genetic counseling, customized recommendations for exercise, nutrition, vascular risk, sleep, cognitive training, stress, and general medical care (199). Cognitive improvement was observed in participants from both higher and lower compliance (199). The results indicate that this approach is promising for improving cognition and reducing AD risk and highlights the importance of multidomain trials for dementia prevention.

## 7. Future directions

Despite the mixed evidence reported in the literature, the benefits of a nutrient-rich diet outweigh the risks. The studies discussed in this review indicate that several diets, including Mediterranean, MIND, DASH, ketogenic, and plant-based, are beneficial for optimal brain health. In addition, intermittent fasting regimes have shown promising results against cognitive decline. Most epidemiological studies indicate these diets’ potential neuroprotective role in preserving cognitive function and possibly preventing dementia. There are several challenges that epidemiological and clinical studies need to overcome. First, the impact of comorbidities, including diabetes, depression, and cardiovascular diseases, should be carefully assessed in the design of these studies. Second, whole foods vs. extracted components should be studied more thoroughly. Third, in addition to genetics and socioeconomic factors, the analysis of sex and gender differences in diet and neuroprotection merits further investigations.

Additionally, other research areas warrant further investigation. For example, the implications of dehydration in the development of MCI and dementia and lithium-containing foods are interesting and promising topics, yet largely unexplored. A multidomain strategy incorporating the six pillars of lifestyle medicine (Figure 1) will be critical for dementia prevention. In this regard, multidimensional clinical trials incorporating a nutrient-rich diet, physical activity and exercise, sleep hygiene, mindfulness and meditation, and management of comorbidities will be key for establishing a successful therapeutic and preventative strategy against cognitive decline and dementia.

## Author contributions

SA, JS, and JP collected the data. SA, MB, JS, and JP wrote the first draft. All authors reviewed and commented on subsequent drafts of the manuscript.

## Funding

This study was funded by the National Institute on Aging (NIA) grant number R01AG062176 to JP. In addition, funds were provided by Rosalind Franklin University of Medicine and Science.

## Conflict of interest

JS is the founder of NeuroHub Analytics, LLC.

The remaining authors declare that the research was conducted in the absence of any commercial or financial relationships that could be construed as a potential conflict of interest.

## Publisher’s note

All claims expressed in this article are solely those of the authors and do not necessarily represent those of their affiliated organizations, or those of the publisher, the editors and the reviewers. Any product that may be evaluated in this article, or claim that may be made by its manufacturer, is not guaranteed or endorsed by the publisher.

## Footnotes

^1^ https://www.healthdata.org/

^2^ https://www.nhlbi.nih.gov/education/dash-eating-plan
